# Age and tumor size as independent predictors of malignancy in BI-RADS 4 and 5 breast lesions: A cross-sectional study in Vietnam

**DOI:** 10.1371/journal.pone.0352690

**Published:** 2026-07-06

**Authors:** De Van Nguyen, Trung Van Pham, Tam Huu Dinh, Dung Ngoc Tran, Chung Thanh Dang

**Affiliations:** 1 Department of Pathology, 108 Military Central Hospital, Hanoi, Viet Nam; 2 Department of Laboratory and Pathology, Military Hospital 7, Haiphong, Vietnam; 3 Department of Pathology and Forensic Medicine, Military Hospital 103, Vietnam Military Medical University, Hanoi, Vietnam; Beth Israel Deaconess Medical Center, UNITED STATES OF AMERICA

## Abstract

**Background:**

Accurate diagnosis of suspicious breast lesions (BI-RADS 4/5) is crucial for appropriate clinical management. The International Academy of Cytology (IAC) Yokohama System standardizes fine-needle aspiration (FNA) cytology reporting, but its performance and the role of independent clinical predictors within this specific high-risk cohort require further investigation. This study aimed to evaluate the diagnostic performance of FNA using the Yokohama system and to identify independent predictors of malignancy in patients with BI-RADS 4 and 5 lesions.

**Methods and findings:**

We conducted a cross-sectional study of 104 female patients with BI-RADS 4/5 breast lesions at two tertiary hospitals in Vietnam. All patients underwent ultrasound-guided FNA with subsequent histopathological examination as the gold standard. Diagnostic performance metrics were calculated. Univariate and multivariate logistic regression analyses were performed to identify independent predictors of malignancy. Of the 104 lesions, 53 (51.0%) were confirmed to be malignant. FNA cytology, interpreted with the Yokohama System, demonstrated a sensitivity of 92.5%, specificity of 96.1%, and an overall accuracy of 94.2%. In the multivariate analysis, only two factors emerged as strong, independent predictors of malignancy: older age (adjusted odds ratio [aOR] 1.13 per year, 95% CI: 1.07–1.19; p < 0.001) and larger tumor size (aOR 1.10 per mm, 95% CI: 1.04–1.16; p = 0.001). Notably, the predictive value of a BI-RADS 5 classification was not statistically significant after this adjustment (p = 0.540). However, given the small BI-RADS 5 subgroup (n = 11), this non-significant result should not be interpreted as evidence of its lack of clinical utility, as the analysis was likely underpowered for this specific variable.

**Conclusion:**

FNA cytology, when interpreted using the IAC Yokohama System, is a highly accurate and reliable tool for the initial evaluation of BI-RADS 4 and 5 breast lesions. Importantly, our findings demonstrate that older age and larger tumor size are strong, independent predictors of malignancy in this high-risk cohort, highlighting the need to integrate these clinical parameters to optimize risk stratification.

## Introduction

Breast cancer remains a significant global health burden and is the leading cause of cancer-related incidence among women worldwide and in Vietnam [1]. The cornerstone of effective management is accurate and timely diagnosis, which relies on the “triple assessment” approach: clinical examination, imaging, and pathological evaluation [2]. Within this framework, ultrasound is a critical imaging modality, and the Breast Imaging Reporting and Data System (BI-RADS) is universally used to standardize risk stratification [3]. Lesions classified as BI-RADS 4 (suspicious) and 5 (highly suggestive of malignancy) represent a particularly challenging cohort, requiring definitive pathological confirmation.

Fine-needle aspiration (FNA) cytology is a minimally invasive, rapid, and cost-effective procedure widely utilized for the initial pathological assessment of these suspicious lesions [4,5]. However, variability in cytological interpretation has historically been a limitation. To address this, the International Academy of Cytology (IAC) developed the Yokohama System, a standardized five-tiered reporting framework that categorizes breast cytology findings and links them to a specific risk of malignancy (ROM), thereby guiding clinical management [6].

While the diagnostic accuracy of the IAC Yokohama System has been validated in general populations [7], a critical knowledge gap remains. Its diagnostic precision within the challenging, pre-selected cohort of BI-RADS 4 and 5 lesions – where the pre-test probability of malignancy is already high – is less understood. This distinction is crucial, as a tool’s utility can differ significantly in a screening population versus a high-risk, diagnostic population. Furthermore, it is crucial to understand the interplay of other clinical and imaging factors that may independently predict malignancy in these patients, as this could help refine diagnostic pathways beyond cytology alone. Identifying robust, independent predictors could empower clinicians to better counsel patients on their individualized risk and to make more informed decisions-for instance, justifying an immediate excisional biopsy over a core needle biopsy in a patient with multiple high-risk features [8].

To address these gaps, this study aimed to: (1) evaluate the diagnostic performance of FNA cytology using the IAC Yokohama System in patients with breast lesions classified as BI-RADS 4 and 5; and (2) identify the independent clinical and imaging predictors of malignancy within this specific high-risk population.

## Methods

### Study design and population

This study was designed, conducted, and reported in accordance with the Strengthening the Reporting of Observational Studies in Epidemiology (STROBE) guidelines (S1 Checklist).

We conducted a cross-sectional study, incorporating both retrospective and prospective data collection, was conducted at the Departments of Pathology of two major tertiary referral hospitals in Hanoi, Vietnam: the Military Hospital 103 and the 108 Military Central Hospital. We reviewed cases from January 2021 to September 2023.

The study population included female patients who presented with a breast lesion classified as BI-RADS category 4 or 5 on ultrasound examination, according to the American College of Radiology (ACR) BI-RADS Atlas [3]. Inclusion criteria were: (1) having undergone both ultrasound-guided FNA and subsequent histopathological examination (either core needle biopsy or excisional biopsy); and (2) having complete clinical records and available pathology slides for review. Patients with recurrent breast cancer or metastatic cancer to the breast from another primary site were excluded. A total of 104 patients with 104 corresponding lesions met the criteria and were included in the final analysis.

### Procedures and pathological evaluation

All patients underwent ultrasound-guided FNA performed by experienced radiologists or pathologists. The aspirated material was smeared onto glass slides, air-dried for Diff-Quik staining, and/or wet-fixed in 95% ethanol for Papanicolaou staining. The cytology slides were reviewed and classified by at least two pathologists according to the five-tiered International Academy of Cytology (IAC) Yokohama System [6]. Any discordant cases were resolved by consensus discussion or, if needed, adjudicated by a third senior pathologist.

The corresponding histopathological specimens were fixed in 10% neutral buffered formalin, processed, and embedded in paraffin. Sections were cut and stained with hematoxylin and eosin (H&E). The final histopathological diagnosis served as the gold standard and was categorized as either benign or malignant based on the 2019 World Health Organization (WHO) Classification of Breast Tumours [9].

### Statistical analysis

Continuous variables were presented as mean ± standard deviation (SD) and compared using the independent samples t-test. Categorical variables were presented as frequencies and percentages (n, %) and compared using the Chi-square test or Fisher’s exact test, as appropriate. The 95% confidence intervals (CIs) for diagnostic performance metrics and malignancy risks were calculated using the Wilson score interval method, which is suitable for small sample sizes.

To identify independent predictors of malignancy, univariate and multivariate logistic regression analyses were performed. The dependent variable was the final histopathological diagnosis (0 = Benign, 1 = Malignant). Independent variables included patient age (continuous), tumor size in millimeters (continuous), and BI-RADS classification (4 vs. 5). Variables for the multivariate model were selected based on their statistical significance in the univariate analysis (p < 0.05) and their established clinical relevance in breast cancer risk assessment. Odds ratios (OR), adjusted odds ratios (aOR), and their corresponding 95% confidence intervals (CI) were calculated.

A post-hoc power calculation was performed based on the observed effect sizes (Cohen’s d) derived from the differences in means between the malignant and benign groups. For tumor size (observed d ≈ 0.64), the analysis yielded a statistical power of 89.5% at a significance level of 0.05. For patient age (observed d ≈ 0.48), the calculated power was 67.2%.

A two-sided *p*-value < 0.05 was considered statistically significant. All statistical analyses were performed using SPSS Statistics for Windows, Version 22.0 (IBM Corp., Armonk, NY, USA).

The primary analysis classified Categories IV (Suspicious for Malignancy) and V (Malignant) as test-positive, and Categories II (Benign) and III (Atypical) as test-negative, consistent with the IAC Yokohama System framework, in which Category III carries a low risk of malignancy (ROM = 6.7%; 95% CI: 0.2–31.9) and is recommended for follow-up or repeat FNA rather than immediate surgical intervention. Two pre-specified sensitivity analyses were performed to assess the robustness of this classification scheme (S4 Table).

### Ethical considerations

This study was conducted in accordance with the principles of the Declaration of Helsinki [10]. The study protocol was reviewed and approved by the Institutional Review Board of the 103 Military Hospital (Decision No. 2030/HĐĐĐ, dated June 23, 2022). Data for this retrospective analysis were accessed from July 2022 to September 2023. As the study involved the analysis of existing clinical and pathological data, the requirement for individual patient consent was waived by the review board. All patient-identifying information was anonymized prior to analysis to ensure confidentiality.

## Results

### Patient and lesion characteristics

A total of 104 female patients with 104 breast lesions classified as BI-RADS 4 or 5 were included. The final histopathological diagnosis confirmed malignancy in 53 lesions (51.0%) and benign findings in 51 (49.0%).

As summarized in Table 1, patients with malignant lesions were significantly older (*p* = 0.011) and presented with larger tumors compared to those with benign lesions (*p* = 0.003 and *p* = 0.042, respectively). The upper-outer quadrant was the most common tumor location (46.2%). The specific histopathological diagnoses for all lesions, including individual benign entities and a distinction between in situ and invasive malignancies, are provided in S3 Table.

**Table 1:** Baseline characteristics of patients and lesions stratified by final histopathological diagnosis.

Characteristic	Benign (n = 51)	Malignant (n = 53)	Total (n = 104)	p-value
**Age (years), mean ± SD**	43.9 ± 16.5	51.2 ± 14.1	47.6 ± 15.6	**0.011** ^a^
**Lesion Size (mm), mean ± SD**	17.3 ± 9.8	25.2 ± 14.5	21.4 ± 12.9	**0.003** ^a^
**Lesion Size Category, n (%)**				**0.042** ^b^
≤ 10 mm, n (%)	11 (21.6)	4 (7.5)	15 (14.4)	
> 10 mm, n (%)	40 (78.4)	49 (92.5)	89 (85.6)	
**BI-RADS Classification, n (%)**				**0.005** ^b^
BI-RADS 4	50 (98.0)	43 (81.1)	93 (89.4)	
BI-RADS 5	1 (2.0)	10 (18.9)	11 (10.6)	
**Tumor Location, n (%)**				**0.033** ^b^
Upper-outer	25 (49.0)	23 (43.4)	48 (46.2)	
Upper-inner	16 (31.4)	9 (17.0)	25 (24.0)	
Lower-outer	8 (15.7)	8 (15.1)	16 (15.4)	
Lower-inner	2 (3.9)	9 (17.0)	11 (10.6)	
Areolar/Nipple	0 (0.0)	4 (7.5)	4 (3.8)	

SD: Standard Deviation; BI-RADS: Breast Imaging Reporting and Data System. (a) independent t-test; (b) Chi-square test or Fisher’s exact test.

### Cytological diagnosis and diagnostic performance

**Fig 1:**
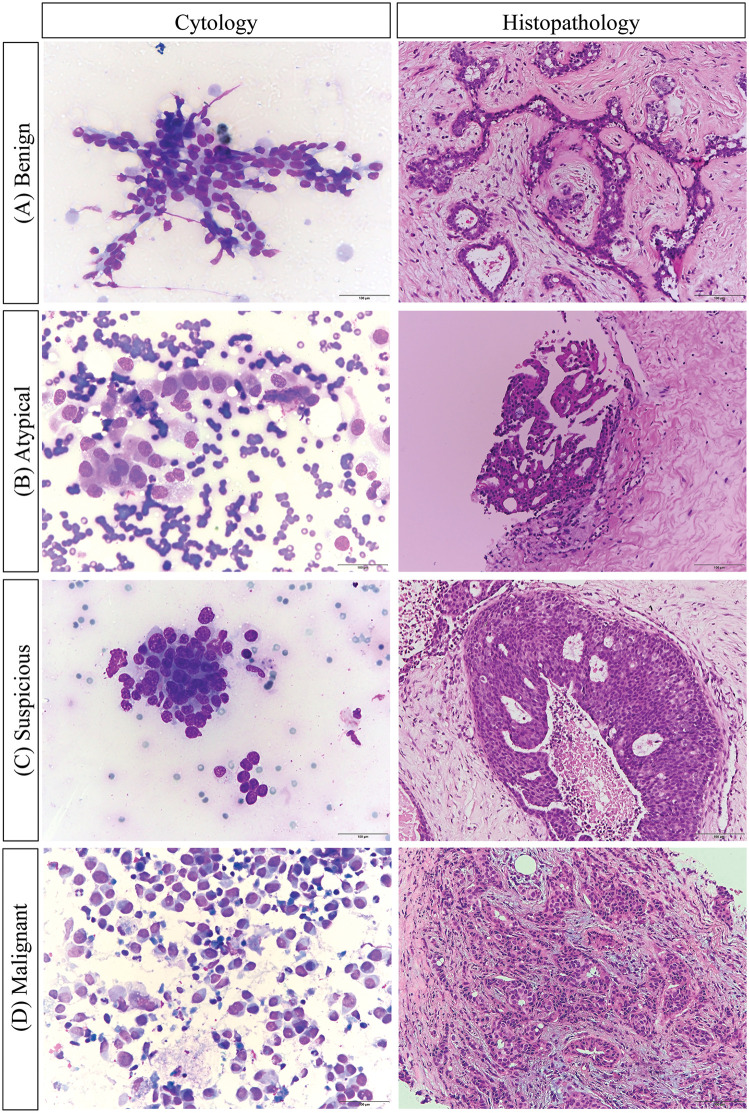
Representative cytological and corresponding histopathological images. (A) Benign (Category II): A large, cohesive, branching sheet of ductal epithelial cells with scattered myoepithelial cells. The corresponding histology shows a fibroadenoma with compressed ducts and fibrous stroma. (B) Atypical (Category III): A moderately cellular smear showing discohesive ductal cells with mild nuclear pleomorphism and prominent nucleoli. The corresponding histology reveals an intraductal papilloma. (C) Suspicious for Malignancy (Category IV): A crowded, three-dimensional cluster of ductal cells with enlarged, overlapping nuclei and irregular nuclear contours. The corresponding histology shows ductal carcinoma in situ with a cribriform pattern. (D) Malignant (Category V): A highly cellular smear composed of discohesive, pleomorphic malignant cells with scant cytoplasm and coarse chromatin. The corresponding histology confirms invasive carcinoma of no special type, characterized by nests and cords of tumor cells infiltrating a desmoplastic stroma. *Cytology panels (left column) are Diff-Quik stained; histopathology panels (right column) are H&E stained. Scale bar = 100 µm.*

Cytological analysis using the IAC Yokohama System classified 49.0% of lesions as either suspicious for malignancy (Category IV, 45.2%) or malignant (Category V, 3.8%). The distribution across all categories is detailed in Table 2, which also shows the risk of malignancy (ROM) for each category. The ROM for Category IV and V were 95.7% and 100%, respectively. Key cytological features observed in malignant cases included enlarged/pleomorphic nuclei (79.2%), absent or rare myoepithelial cells (75.5%), and prominent nucleoli (56.6%) (Fig 1).

**Table 2:** Correlation between fine-needle aspiration cytology (IAC Yokohama System) and final histopathology.

IAC Yokohama Category	Histopathology: Benign (n = 51)	Histopathology: Malignant (n = 53)	Total (n = 104)	Risk of Malignancy % (95% CI)
**II: Benign**	35	3	38	7.9 (1.7–21.4)
**III: Atypical**	14	1	15	6.7 (0.2–31.9)
**IV: Suspicious for Malignancy**	2	45	47	95.7 (85.5–99.5)
**V: Malignant**	0	4	4	100.0 (51.0–100.0)

IAC: International Academy of Cytology; CI: Confidence Interval. Risk of Malignancy calculated as (Malignant cases/ Total cases in category) × 100. 95% CIs were calculated using the Wilson score interval method.

For the purpose of calculating diagnostic performance, Categories IV and V were considered positive results. The overall sensitivity of FNA was 92.5% (95% CI: 82.1–97.9) and specificity was 96.1% (95% CI: 86.5–99.5). The positive and negative predictive values were 96.1% and 92.5%, respectively. The overall accuracy was 94.2% (Table 3). Results were consistent across two pre-specified sensitivity analyses; full details are provided in S4 Table.

**Table 3:** Diagnostic performance of fine-needle aspiration cytology for malignancy.

Diagnostic Metric	Value (%)	95% Confidence Interval
**Sensitivity**	92.5	82.1–97.9
**Specificity**	96.1	86.5–99.5
**Positive Predictive Value (PPV)**	96.1	86.5–99.5
**Negative Predictive Value (NPV)**	92.5	82.1–97.9
**Accuracy**	94.2	88.1–97.8

Categories IV (Suspicious) and V (Malignant) were classified as positive; Categories II (Benign) and III (Atypical) were classified as negative.

### Independent predictors of malignancy

While univariate analysis identified older age, larger tumor size, and a BI-RADS 5 classification as significant predictors of malignancy, only two factors retained statistical significance in the multivariate model. Older age (aOR 1.13, 95% CI: 1.07–1.19; *p* < 0.001) and larger tumor size (aOR 1.10, 95% CI: 1.04–1.16; *p* = 0.001) emerged as strong and independent predictors. The predictive value of a BI-RADS 5 classification was not statistically significant after adjusting for age and tumor size (*p* = 0.540) (Table 4).

**Table 4:** Univariate and multivariate logistic regression analysis of predictors for malignancy.

Predictor Variable	Crude OR (95% CI)	p-value	Adjusted aOR (95% CI)	p-value
**Age** *(per 1-year increase)*	1.10 (1.06–1.15)	<0.001	1.13 (1.07–1.19)	**<0.001**
**Tumor Size** *(per 1-mm increase)*	1.06 (1.02–1.10)	0.006	1.10 (1.04–1.16)	**0.001**
**BI-RADS 5** *(vs. BI-RADS 4)*	11.63 (1.43–94.54)	0.022	2.10 (0.20–22.73)	0.540

OR, Odds Ratio; aOR, Adjusted Odds Ratio; CI, Confidence Interval; BI-RADS, Breast Imaging Reporting and Data System. The adjusted odds ratios were derived from a single multivariate logistic regression model that included age, tumor size, and BI-RADS classification simultaneously.

## Discussion

This study confirms the high diagnostic utility of fine-needle aspiration (FNA) cytology, when applying the IAC Yokohama System, for risk stratification of suspicious breast lesions classified as BI-RADS 4 and 5. Our key findings demonstrate that while FNA provides excellent sensitivity and specificity, the most robust independent predictors of malignancy in this high-risk cohort are older age and larger tumor size. Notably, the BI-RADS 5 classification-while highly predictive on its own-lost its independent predictive power after adjusting for these two fundamental clinical variables.

The overall diagnostic performance of FNA in our study (sensitivity 92.5%, specificity 96.1%) is consistent with the high accuracy reported in a growing body of international literature. A comprehensive review by Hoda et al. reported a pooled sensitivity and specificity of 96.3% and 98.8% [7], and our results closely mirror findings from large institutional studies in Italy, India, and the United States, confirming the system’s robust reproducibility across diverse populations [11–14].

The risk of malignancy (ROM) for each cytological category in our cohort also aligns with established benchmarks. The ROM for our ‘Suspicious for Malignancy’ (Category IV) and ‘Malignant’ (Category V) groups were 95.7% and 100%, respectively. These findings reinforce that such cytological results are highly indicative of cancer and warrant definitive histopathological examination, a conclusion supported by multiple validation studies [11,15]. This underscores the system’s reliability and applicability within the Vietnamese clinical context for guiding patient management.

The most significant finding of our study emerged from the multivariate analysis. While older age and larger tumor size are well-established risk factors for breast cancer [16,17], our model demonstrates they are powerful independent predictors that can overshadow the predictive value of BI-RADS classification in a pre-selected high-risk population.

The finding that BI-RADS 5 lost its statistical significance (p = 0.540) after adjusting for age and tumor size is particularly intriguing. This suggests that the morphological features on ultrasound that lead to a BI-RADS 5 classification (e.g., spiculation, irregular shape, posterior acoustic shadowing) may be biologically linked to the characteristics of tumors that develop in older women or that have grown to a larger size. Indeed, studies have shown that tumor size is a strong determinant of sonographic features, with larger tumors more likely to exhibit malignant characteristics [18–20].

Therefore, it is plausible that in a multivariate context, age and size act as more fundamental proxies for the underlying tumor biology. The sonographic features defining BI-RADS 5 may simply be the morphological manifestations of tumors that have had more time to grow (larger size) in a host environment conducive to progression (older age), thereby making age and size the more direct measures of malignant potential. This phenomenon, where clinical or pathological variables can attenuate the significance of imaging scores, highlights the importance of multi-faceted risk assessment [18,21,22].

Our study has several strengths. It is one of the first in Vietnam to rigorously evaluate the Yokohama system within a specific, clinically relevant cohort of BI-RADS 4 and 5 lesions. Furthermore, the use of multivariate logistic regression provides a deeper, more nuanced understanding of the interplay between clinical, imaging, and cytological factors.

However, we must also acknowledge certain limitations, as recommended by the STROBE guidelines [18,23]. First, the study was conducted at two major tertiary centers, which may limit the generalizability of our findings to provincial or community-level healthcare settings. Second, our sample size (n = 104), particularly for the BI-RADS 5 subgroup (n = 11), is relatively small. Retroactive expansion of the dataset was not feasible due to our strict inclusion criteria requiring matched high-quality FNA and definitive histopathology to ensure diagnostic accuracy. A post-hoc power analysis indicated that while the study achieved robust statistical power for tumor size (89.5%), the power for patient age was moderate (67.2%), and the BI-RADS 5 subgroup remained underpowered. This limitation resulted in wide confidence intervals for BI-RADS 5 in the multivariate model, raising the possibility of a Type II error for this specific variable [24]. Consequently, findings regarding the loss of independent predictive value for BI-RADS 5 should be interpreted as preliminary. Third, although cytological slides were reviewed by two pathologists with a consensus approach for discordant cases, potential interobserver variability remains an inherent limitation of morphological assessment. Fourth, subcategory data for BI-RADS 4 lesions (4A, 4B, and 4C) were unavailable, as structured subcategory reporting was not consistently documented in the institutional radiology records during the retrospective data collection period (2021–2023). This limits the granularity of the BI-RADS analysis, as the three BI-RADS 4 subcategories carry substantially different risks of malignancy (4A: ≥ 2% to <10%; 4B: ≥ 10% to ≤50%; 4C: > 50% to <95%). Future prospective studies should incorporate BI-RADS 4 subcategory classification to enable a more refined analysis of imaging-based malignancy predictors within this heterogeneous group.

Our findings have important clinical implications. FNA cytology based on the Yokohama system is a robust and reliable tool for the initial assessment of high-risk breast lesions in Vietnam. Clinicians and pathologists should recognize that for lesions already classified as BI-RADS 4 or 5, patient age and tumor size are potent, independent indicators of malignancy. This finding suggests that in resource-limited settings where further molecular testing may not be immediately available, these readily accessible clinical parameters can significantly aid in counseling patients and prioritizing cases for expedited biopsy or surgical consultation. This highlights the necessity of integrating clinical data with imaging and cytology for the most accurate risk assessment, a principle supported by major clinical guidelines such as those from the NCCN and ASCO [25,26]. Future research should include larger, multi-center prospective studies to validate our findings on the predictive roles of these variables and explore their molecular profiles [27].

## Conclusion

In patients with suspicious breast lesions classified as BI-RADS 4 and 5, fine-needle aspiration cytology evaluated by the IAC Yokohama System is a highly accurate and reliable diagnostic tool. Importantly, our findings demonstrate that older age and larger tumor size are strong, independent predictors of malignancy in this high-risk cohort. These results suggest that in resource-limited settings like Vietnam, where molecular testing may not be immediately available, integrating these readily accessible clinical parameters with imaging and cytology is essential for optimizing risk stratification. Of note, the non-significant result for BI-RADS 5 in the multivariate model (p = 0.540; aOR 2.10, 95% CI: 0.20–22.73) should not be interpreted as evidence that BI-RADS 5 classification lacks clinical value; rather, it reflects the limited statistical power of the small BI-RADS 5 subgroup (n = 11), which rendered this estimate susceptible to Type II error. This approach can help clinicians prioritize patients for expedited biopsy or surgical intervention, ensuring timely and appropriate management.

## Supporting information

S1 Checklist STROBE Checklist. (DOC)

S2 Dataset Anonymized dataset; Age column hidden. No personal identifiers present.
(XLSX)

S3 Table Histopathological diagnoses of 104 breast lesions classified according to the WHO Classification of Breast Tumours (2019). (DOCX)

S4 Table Sensitivity analyses for diagnostic performance of FNA cytology under alternative Category III classification schemes. (DOCX)
