# Supplementary material for: Age and tumor size as independent predictors of malignancy in BI-RADS 4 and 5 breast lesions: A cross-sectional study in Vietnam
Source: PLoS One. 2026 Jul 6;21(7):e0352690. doi: 10.1371/journal.pone.0352690 (PMC13336213; doi:10.1371/journal.pone.0352690)
Supplement: S1 Checklist — (DOC) [file pone.0352690.s001.doc]

STROBE Statement—Checklist of items that should be included in reports of ***cross-sectional studies***

|  | Item No | Recommendation | Location in Manuscript (Section and/or Page Number) |
| --- | --- | --- | --- |
| **Title and abstract** | 1 | (*a*) Indicate the study’s design with a commonly used term in the title or the abstract | Title (Page 1): "...A cross-sectional study in Vietnam"; Abstract (Page 2): "We conducted a cross-sectional study..." |
|  |  | (*b*) Provide in the abstract an informative and balanced summary of what was done and what was found | Abstract (Page 2): Provides structured summary of background, methods, results (sensitivity, specificity, aORs), and conclusions. |
| Introduction | | |  |
| Background/rationale | 2 | Explain the scientific background and rationale for the investigation being reported | Introduction (Page 3-4): Paragraphs 1-3 detail the burden of breast cancer, triple assessment, and the IAC Yokohama System. |
| Objectives | 3 | State specific objectives, including any prespecified hypotheses | Introduction (Page 4): Paragraph 4 states aim to evaluate FNA performance and identify independent predictors of malignancy. |
| Methods | | |  |
| Study design | 4 | Present key elements of study design early in the paper | Methods (Page 5): Study design and population, Paragraph 1. |
| Setting | 5 | Describe the setting, locations, and relevant dates, including periods of recruitment, exposure, follow-up, and data collection | Methods (Page 5): Study design and population, Paragraph 1. Data collection: January 2021 to September 2023. |
| Participants | 6 | (*a*) Give the eligibility criteria, and the sources and methods of selection of participants | Methods (Page 5): Study design and population, Paragraph 2. Lists inclusion/exclusion criteria |
| Variables | 7 | Clearly define all outcomes, exposures, predictors, potential confounders, and effect modifiers. Give diagnostic criteria, if applicable | Methods (Page 5-6): Statistical analysis (defines predictors and outcomes); Procedures and Pathological Evaluation (defines diagnostic criteria). |
| Data sources/ measurement | 8* | For each variable of interest, give sources of data and details of methods of assessment (measurement). Describe comparability of assessment methods if there is more than one group | Methods (Page 5-6): Procedures and Pathological Evaluation. Details on FNA and histopathological examination. |
| Bias | 9 | Describe any efforts to address potential sources of bias | Methods (Page 5-6): Procedures and Pathological Evaluation (consensus diagnosis). Discussion (Page 14): Discusses selection bias and interobserver variability. |
| Study size | 10 | Explain how the study size was arrived at | Methods (Page 5): Study Design and Population, Paragraph 2. Methods (Page 6): Statistical Analysis (Post-hoc power analysis added) |
| Quantitative variables | 11 | Explain how quantitative variables were handled in the analyses. If applicable, describe which groupings were chosen and why | Methods (Page 6): Statistical Analysis. Details handling of continuous (mean ± SD) and categorical variables. |
| Statistical methods | 12 | (*a*) Describe all statistical methods, including those used to control for confounding | Methods (Page 6-7): Statistical Analysis. Details t-test, Chi-square, Wilson score interval for CIs, and logistic regression. |
|  |  | (*b*) Describe any methods used to examine subgroups and interactions | Not applicable. No subgroup or interaction analyses were performed. |
|  |  | (*c*) Explain how missing data were addressed | Methods, Study Design and Population, Paragraph 2: Complete records were part of the inclusion criteria, thus there was no missing data for the analyzed variables. |
|  |  | (*d*) If applicable, describe analytical methods taking account of sampling strategy | Not applicable. The study included all eligible consecutive patients. |
|  |  | (*e*) Describe any sensitivity analyses | Not applicable. No sensitivity analyses were performed. |
| Results | | |  |
| Participants | 13* | (a) Report numbers of individuals at each stage of study—eg numbers potentially eligible, examined for eligibility, confirmed eligible, included in the study, completing follow-up, and analysed | Results (Page 8): Patient and Lesion Characteristics, Paragraph 1. |
|  |  | (b) Give reasons for non-participation at each stage | Not applicable. The study reports on the final cohort that met all inclusion criteria. |
|  |  | (c) Consider use of a flow diagram | A flow diagram was not used, but the selection process is described in Methods, Study Design and Population, Paragraph 2. |
| Descriptive data | 14* | (a) Give characteristics of study participants (eg demographic, clinical, social) and information on exposures and potential confounders | Results (Page 8-9): Table 1 presents baseline clinical and demographic characteristics. |
|  |  | (b) Indicate number of participants with missing data for each variable of interest | Not applicable. As stated in Methods, complete records were required for inclusion. |
| Outcome data | 15* | Report numbers of outcome events or summary measures | Results (Page 8): Patient and Lesion Characteristics, Paragraph 1 (53 malignant and 51 benign cases). |
| Main results | 16 | (*a*) Give unadjusted estimates and, if applicable, confounder-adjusted estimates and their precision (eg, 95% confidence interval). Make clear which confounders were adjusted for and why they were included | Results (Page 10-11): Table 3 (diagnostic metrics) and Table 4 (Crude and Adjusted Odds Ratios with 95% CIs). |
|  |  | (*b*) Report category boundaries when continuous variables were categorized | Results (Page 8): Table 1 shows categorization for Lesion Size (≤10 mm vs >10 mm) |
|  |  | (*c*) If relevant, consider translating estimates of relative risk into absolute risk for a meaningful time period | Not applicable. No other analyses were reported. |
| Other analyses | 17 | Report other analyses done—eg analyses of subgroups and interactions, and sensitivity analyses | Methods (Page 6-7): Statistical Analysis. A post-hoc power analysis was performed to evaluate the statistical power for tumor size and patient age. Results (Page 14): Discussion section addresses the implications of the power analysis for the BI-RADS 5 subgroup |
| Discussion | | |  |
| Key results | 18 | Summarise key results with reference to study objectives | Discussion (Page 12): Paragraph 1 summarizes the findings in relation to study objectives |
| Limitations | 19 | Discuss limitations of the study, taking into account sources of potential bias or imprecision. Discuss both direction and magnitude of any potential bias | Discussion (Page 14-15): Paragraph 4 discusses selection bias, small subgroup size, and interobserver variability. |
| Interpretation | 20 | Give a cautious overall interpretation of results considering objectives, limitations, multiplicity of analyses, results from similar studies, and other relevant evidence | Discussion (Page 12-15): Results are compared with international literature and clinical implications are discussed. |
| Generalisability | 21 | Discuss the generalisability (external validity) of the study results | Discussion (Page 14): Paragraph 8 addresses limitations of tertiary center settings. |
| Other information | | |  |
| Funding | 22 | Give the source of funding and the role of the funders for the present study and, if applicable, for the original study on which the present article is based | To be declared in the submission system. The manuscript states "The author(s) received no specific funding for this work." |

*Give information separately for exposed and unexposed groups.

**Note:** An Explanation and Elaboration article discusses each checklist item and gives methodological background and published examples of transparent reporting. The STROBE checklist is best used in conjunction with this article (freely available on the Web sites of PLoS Medicine at http://www.plosmedicine.org/, Annals of Internal Medicine at http://www.annals.org/, and Epidemiology at http://www.epidem.com/). Information on the STROBE Initiative is available at www.strobe-statement.org.
