# Supplementary material for: Age and tumor size as independent predictors of malignancy in BI-RADS 4 and 5 breast lesions: A cross-sectional study in Vietnam
Source: PLoS One. 2026 Jul 6;21(7):e0352690. doi: 10.1371/journal.pone.0352690 (PMC13336213; doi:10.1371/journal.pone.0352690)
Supplement: S3 Table — (DOCX) [file pone.0352690.s003.docx]

**S3 Table. Histopathological diagnoses of 104 breast lesions classified according to the WHO Classification of Breast Tumours (2019).**

| **WHO 2019 Category**  **Histopathological Diagnosis** | | **n** | **% of subgroup** | **% of total (n = 104)** |
| --- | --- | --- | --- | --- |
| **A. Benign lesions** | | **51** | **100.0** | **49.0** |
|  | Fibroadenoma | 37 | 72.5 | 35.6 |
|  | Fibrocystic change | 3 | 5.9 | 2.9 |
|  | Intraductal papilloma | 3 | 5.9 | 2.9 |
|  | Mastitis / Inflammatory lesion | 3 | 5.9 | 2.9 |
|  | Phyllodes tumour | 1 | 2.0 | 1.0 |
|  | Sclerosing adenosis | 2 | 3.9 | 1.9 |
|  | Epithelial hyperplasia, usual type² | 2 | 3.9 | 1.9 |
| **B. Malignant lesions** | | **53** | **100.0** | **51.0** |
| *B1. Non-invasive* | |  | | |
|  | Ductal carcinoma in situ (DCIS), NOS | 1 | 1.9 | 1.0 |
| *B2. Invasive* | |  | | |
|  | Invasive carcinoma of no special type (NST)³ | 47 | 88.7 | 45.2 |
|  | Tubular carcinoma | 4 | 7.5 | 3.8 |
|  | Mucinous carcinoma | 1 | 1.9 | 1.0 |
| **Total** | | **104** |  | **100.0** |

*^Abbreviations:^ DCIS = ductal carcinoma in situ; NOS = not otherwise specified; NST = no special type; WHO = World Health Organization.
^1^ Classification per WHO Classification of Breast Tumours, 4th Edition. Lyon: International Agency for Research on Cancer (IARC), 2019. ISBN 978-92-832-4500-1.
^2^ Usual-type epithelial hyperplasia (WHO 2019): non-atypical proliferation of ductal epithelium; excluded from the atypical ductal hyperplasia category.
^3^ Includes one case of invasive solid carcinoma, a morphological pattern of invasive carcinoma of no special type (NST) per WHO 2019.
Percentages are rounded to one decimal place and may not sum to 100% due to rounding.*
