# Supplementary material for: Age and tumor size as independent predictors of malignancy in BI-RADS 4 and 5 breast lesions: A cross-sectional study in Vietnam
Source: PLoS One. 2026 Jul 6;21(7):e0352690. doi: 10.1371/journal.pone.0352690 (PMC13336213; doi:10.1371/journal.pone.0352690)
Supplement: S4 Table — (DOCX) [file pone.0352690.s004.docx]

**S4 Table. Sensitivity analyses for the diagnostic performance of fine-needle aspiration cytology (IAC Yokohama System) under alternative Category III classification schemes.**

| **Diagnostic Metric** | **Main Analysis (n = 104) Cat IV+V = Positive** | **Sensitivity Analysis 1 (n = 89, Cat III excluded) Cat IV+V = Positive** | **Sensitivity Analysis 2 (n = 104) Cat III+IV+V = Positive** |
| --- | --- | --- | --- |
| ***2×2 Contingency Matrix*** |  |  |  |
| True Positive (TP) | 49 | 49 | 50 |
| False Positive (FP) | 2 | 2 | 16 |
| False Negative (FN) | 4 | 3 | 3 |
| True Negative (TN) | 49 | 35 | 35 |
| ***Diagnostic Performance*** |  |  |  |
| Sensitivity, % (95% CI) | 92.5 (82.1-97.9) | 94.2 (84.4-98.0) | 94.3 (84.6-98.1) |
| Specificity, % (95% CI) | 96.1 (86.5-99.5) | 94.6 (82.3-98.5) | **68.6** (55.0-79.7) |
| PPV, % (95% CI) | 96.1 (86.5-99.5) | 96.1 (86.8-98.9) | **75.8** (64.2-84.5) |
| NPV, % (95% CI) | 92.5 (82.1-97.9) | 92.1 (79.2-97.3) | 92.1 (79.2-97.3) |
| Accuracy, % (95% CI) | 94.2 (88.1-97.8) | 94.4 (87.5-97.6) | **81.7** (73.2-88.0) |

*Abbreviations: TP, true positive; FP, false positive; FN, false negative; TN, true negative; PPV, positive predictive value; NPV, negative predictive value; CI, confidence interval; Cat, Category; IAC, International Academy of Cytology.*

*Main Analysis: Categories IV (Suspicious for Malignancy) and V (Malignant) were classified as test-positive; Categories II (Benign) and III (Atypical) were classified as test-negative. This classification is consistent with the IAC Yokohama System original framework, in which Category III (ROM = 6.7%; 95% CI: 0.2-31.9) carries a low risk of malignancy and is recommended for follow-up or repeat FNA rather than immediate surgical intervention (Field et al., Acta Cytol 2019;63:257-273).*

*Sensitivity Analysis 1 (Cat III excluded, n = 89): The 15 Category III cases were excluded from the analysis to assess diagnostic performance in cytologically unambiguous cases only. This represents the most conservative scenario.*

*Sensitivity Analysis 2 (Cat III = Positive, n = 104): All 15 Category III cases were reclassified as test-positive (low-threshold scenario). This approach substantially reduces specificity (68.6%) and PPV (75.8%) - highlighted in yellow - reflecting the clinical cost of treating atypical cytology as an actionable positive result.*

*95% CIs were calculated using the Wilson score interval method.*
