# Supplementary material for: Age and tumor size as independent predictors of malignancy in BI-RADS 4 and 5 breast lesions: A cross-sectional study in Vietnam
Source: PLoS One. 2026 Jul 6;21(7):e0352690. doi: 10.1371/journal.pone.0352690 (PMC13336213; doi:10.1371/journal.pone.0352690)
Supplement: Attachment — Submitted filename: Response to Reviewers.doc [file pone.0352690.s005.doc]

January 26, 2026

**Ref: PONE-D-25-49862** **Title:** Age and tumor size as independent predictors of malignancy in BI-RADS 4 and 5 breast lesions: A cross-sectional study in Vietnam

Dear Dr. Dongling Wu and Reviewers,

We would like to express our sincere gratitude for the time and effort you have dedicated to reviewing our manuscript. We appreciate the insightful comments and constructive suggestions, which have helped us significantly improve the quality and rigor of our study.

We have carefully reviewed all points raised. In this revised version, we have addressed the concerns regarding sample size by providing a post-hoc power analysis and clarifying our strict inclusion criteria. We have also complied with PLOS ONE’s data policy by uploading a minimal underlying dataset (S2 Dataset).

Below is our point-by-point response to the specific comments.

**RESPONSE TO JOURNAL REQUIREMENTS**

**1. PLOS ONE Style Requirements**

*Comment: Please ensure that your manuscript meets PLOS ONE's style requirements, including those for file naming.*

**Response:** We have reviewed the PLOS ONE style templates and formatted our manuscript accordingly. Specifically, we have adjusted the headings (Level 1 in 18pt bold), figure citations, and reference style to meet the journal's guidelines.

**2. STROBE Checklist**

*Comment: Please include a copy of S1 Checklist. STROBE Checklist which you refer to in your text.*

**Response:** We have completed the STROBE Checklist with page numbers indicating where each item is addressed in the revised manuscript. This file has been uploaded as S1 Checklist.

**3. Data Availability**

*Comment: “Have the authors made all data underlying the findings in their manuscript fully available?” is not sufficient.*

**Response:** We apologize for the initial restriction. We have now prepared a de-identified minimal dataset containing the values behind the means, standard deviations, and other measures reported. This dataset complies with ethical standards for patient anonymity and has been uploaded as a Supporting Information file labeled ‘S2 Dataset’. The Data Availability Statement within the manuscript has been updated accordingly.

**RESPONSE TO REVIEWER #1**

**Comment 1: Sample size and Statistical power**

*Reviewer #1: The sample size is small (104 lesions) and key subgroups particularly BI-RADS 5 lesions are underpowered as reflected by extremely wide confidence intervals in the multivariate analysis. No sample size justification or power calculation is provided, raising concerns about Type II errors and the reliability of reported associations.*

**Response:** We thank the reviewer for this critical assessment. We agree that the sample size, particularly for the BI-RADS 5 subgroup (n=11), is a limitation. Regarding the suggestion to include more years of data, we evaluated this possibility but found it unfeasible due to our strict inclusion criteria, which required matched high-quality ultrasound-guided FNA and definitive histopathology (biopsy/surgery) for every patient to ensure diagnostic accuracy. Extending the timeframe retrospectively would have compromised data completeness and quality due to administrative limitations on older physical records.

However, to address the concern regarding Type II errors and reliability, we have performed a post-hoc power analysis based on the observed effect sizes in our study (added to *Methods*, Page 6-7, Lines: 113-116)

- For tumor size: The observed effect size was medium-to-large (≈ 0.64). Our analysis indicates the study achieved a statistical power of 89.5% (α=0.05) to detect this primary predictor. This suggests the finding regarding tumor size is robust.
- For patient age: The observed effect size was moderate (≈ 0.48), yielding a power of 67.2%. While slightly below the conventional 80%, the significant association (p = 0.003) suggests a detectable signal in our population.

We have also revised the Discussion section (Pages 14, Lines 219-228) to explicitly acknowledge that the BI-RADS 5 subgroup is underpowered, leading to wide confidence intervals, and that findings regarding this specific subgroup should be interpreted as preliminary.

**Comment 2: Data Availability**

*Reviewer #1: Have the authors made all data underlying the findings in their manuscript fully available? No.*

**Response:** As mentioned in the response to the Journal Requirements, we have now uploaded the fully anonymized minimal dataset as S2 Dataset.

**RESPONSE TO REVIEWER #2**

**Comment 1: Methodology and Writing**

*Reviewer #2: The manuscript is very well written, the methodology is well conducted, and the work is robust. I offer a few recommendations.*

**Response:** We are grateful for the reviewer’s positive evaluation of our work and the helpful recommendations to improve the manuscript’s readability.

**Comment 2: Paragraph Length and Readability**

*Reviewer #2: Some paragraphs (especially in the Discussion) are long and could be made slightly more objective. The manuscript alternates between shorter sentences and some very long ones; a few of these could be broken up to improve readability.*

**Response:** We have reviewed the Discussion section and broken down long paragraphs and complex sentences to improve flow and clarity, as suggested. (See changes on Pages 12, 13, 14).

**Comment 3: Table Clarity**

*Reviewer #2: In Table 1, the positioning of the p-values could be reorganized to make the layout visually cleaner. In Table 2, it may be useful to include the exact ROM values with their confidence intervals, should the authors wish to strengthen the presentation.*

**Response:**

- Table 1: We have reorganized the layout of Table 1. P-values are now presented in a dedicated column on the right side to improve readability and visual alignmen (Pages 8 - 9).
- Table 2: We have calculated the 95% Confidence Intervals (CI) for the Risk of Malignancy (ROM) using the Wilson score interval method (appropriate for small sample sizes). These values have been added to the last column of Table 2 (Page 9).

**Comment 4: Limitations (Tertiary Centers & Interobserver Variability)**

*Reviewer #2: Although the limitations are already well described, the authors could consider adding: the potential bias associated with conducting the study in tertiary centers, and the possibility of interobserver variability in cytology interpretation (even though consensus among pathologists was used).*

**Response:** We agree these are important points.

- We have reinforced the statement regarding the tertiary center bias (Page 14, Lines 218-219).
- We have added a sentence to the Limitations section acknowledging the potential for interobserver variability in cytology interpretation, despite our consensus method (Page 14, Lines 228-230).

**Comment 5: Clinical Applicability**

*Reviewer #2: Finally, consider adding a concluding paragraph that more explicitly reinforces the clinical applicability of the findings.*

**Response:** We have revised the Conclusion to explicitly state the clinical implication: in resource-limited settings like Vietnam, integrating Age and Tumor Size into the risk assessment of BI-RADS 4/5 lesions can help clinicians prioritize patients for urgent intervention. (Page 15, Lines 247-251).

Sincerely,

Assoc. Prof. Dang Thanh Chung, MD, PhD

On behalf of the co-authors

Vice Head, Department of Pathology and Forensic Medicine Military Hospital 103,

Vietnam Military Medical University Hanoi, Vietnam

dangthanhchung@vmmu.edu.vn
