# Supplementary material for: Age and tumor size as independent predictors of malignancy in BI-RADS 4 and 5 breast lesions: A cross-sectional study in Vietnam
Source: PLoS One. 2026 Jul 6;21(7):e0352690. doi: 10.1371/journal.pone.0352690 (PMC13336213; doi:10.1371/journal.pone.0352690)
Supplement: Attachment — Submitted filename: Response_to_Reviewers_auresp_2.doc [file pone.0352690.s006.doc]

May 27, 2026

**Ref: PONE-D-25-49862R1**

*Title: Age and tumor size as independent predictors of malignancy in BI-RADS 4 and 5 breast lesions: A cross-sectional study in Vietnam*

Journal: PLOS ONE

Dear Dr. Dongling Wu and Members of the Editorial Board,

We thank the Academic Editor and Reviewers for their careful evaluation and constructive comments, which have substantially strengthened the revised manuscript. We have addressed all four comments raised by Reviewer 3. A point-by-point response is provided below. All revisions are visible in the Revised Manuscript with Track Changes.

**RESPONSE TO REVIEWER #2**

**Comment***: The authors addressed all comments satisfactorily and improved the manuscript, particularly regarding the clarity of the writing and the transparency of the presented data.*

**Response:** We thank Reviewer 2 for this positive assessment.

**RESPONSE TO REVIEWER #3**

We thank Reviewer 3 for the thorough and methodologically rigorous comments. Responses to each point are provided below, with corresponding changes indicated by line numbers in the Revised Manuscript with Track Changes.

**Comment 1:** *The manuscript would be stronger if the authors provided a table summarizing the histopathologic diagnoses, including benign entities, DCIS, invasive carcinoma of no special type, special histologic types if present, and whether malignant cases were invasive or in situ.*

**Response:** We have prepared Supplementary Table S3 (S3 Table), which provides a complete breakdown of all histopathological diagnoses for the 104 lesions, classified per the WHO Classification of Breast Tumours, 4th Edition (2019). Specifically, S3 Table:

1. Lists all benign entities individually (fibroadenoma, fibrocystic change, intraductal papilloma, mastitis/inflammatory lesion, phyllodes tumour, sclerosing adenosis, and epithelial hyperplasia, usual type), per WHO 2019 nomenclature.
2. Clearly distinguishes non-invasive from invasive malignancies: ductal carcinoma in situ (DCIS), NOS (n = 1) is listed separately under “B1. Non-invasive.”
3. Lists all special histological subtypes of invasive carcinoma: invasive carcinoma of no special type (NST; n = 47, including one case with a solid growth pattern), tubular carcinoma (n = 4), and mucinous carcinoma (n = 1), consistent with WHO 2019 nomenclature.

***Changes in the revised manuscript:***

(1) The following sentence has been added to the Results section, end of the 'Patient and lesion characteristics' paragraph:

*"The specific histopathological diagnoses for all lesions, including individual benign entities and a distinction between in situ and invasive malignancies, are provided in S3 Table."*

(2) S3 Table has been added to the Supporting Information list at the end of the manuscript. No existing text, data, or tables (Tables 1-4) have been modified.

**Comment 2:** *Similarly, if available, BI-RADS 4 should be subdivided into 4A, 4B, and 4C. Without this, the BI-RADS 4 group is heterogeneous, and the clinical meaning of comparing BI-RADS 4 versus BI-RADS 5 is limited.*

**Response:** The suggestion is fully justified: the ACR BI-RADS Atlas (5th ed., 2013) assigns meaningfully different risks of malignancy to each subcategory (4A: ≥2% to <10%; 4B: ≥10% to ≤50%; 4C: >50% to <95%). However, BI-RADS 4 subcategory data (4A/4B/4C) were not available in the source medical records. The study incorporated data from two military tertiary hospitals in Vietnam collected between January 2021 and September 2023, a period during which structured subcategory reporting was not consistently documented in institutional radiology reports. The binary BI-RADS 4 vs. 5 classification therefore reflects the data as recorded in the original clinical records.

We fully acknowledge that the undifferentiated BI-RADS 4 group introduces heterogeneity into the analysis and limits the clinical interpretability of the BI-RADS 4 vs. 5 comparison. This has been explicitly stated as an additional limitation in the revised Discussion section.

***Changes in the revised manuscript:***

The following paragraph has been added as the fourth item in the Limitations section of the Discussion (Track Changes):

*"Fourth, subcategory data for BI-RADS 4 lesions (4A, 4B, and 4C) were unavailable, as structured subcategory reporting was not consistently documented in the institutional radiology records during the retrospective data collection period (2021-2023). This limits the granularity of the BI-RADS analysis, as the three BI-RADS 4 subcategories carry substantially different risks of malignancy (4A: ≥2% to <10%; 4B: ≥10% to ≤50%; 4C: >50% to <95%). Future prospective studies should incorporate BI-RADS 4 subcategory classification to enable a more refined analysis of imaging-based malignancy predictors within this heterogeneous group."*

No data, statistics, or existing conclusions have been modified.

**Comment 3:** *The authors classified Categories IV and V as positive, and Categories II and III as negative for diagnostic performance calculations. This approach may underestimate the clinical uncertainty associated with “atypical” cytology. The authors should justify this decision and ideally provide sensitivity analyses: one excluding Category III cases and another treating Category III as a test-positive or indeterminate result. This would make the reported sensitivity, specificity, PPV, NPV, and accuracy more transparent.*

**Response:** We agree with this assessment and have responded with: (1) a formal justification of the primary classification scheme, incorporated into the Methods section; and (2) two pre-specified sensitivity analyses, presented in S4 Table.

***Part 1 - Justification of the primary classification (Cat IV+V = Positive)***

The decision to classify Categories IV and V as test-positive and Categories II and III as test-negative was made a priori, following the IAC Yokohama System framework (Field AS et al. Acta Cytol. 2019;63:257-273), in which Category III (Atypical) carries a low risk of malignancy and is recommended for repeat FNA or close follow-up rather than immediate surgical intervention. In our cohort, Category III yielded a ROM of 6.7% (95% CI: 0.2-31.9%), which is statistically comparable to Category II (ROM 7.9%; 95% CI: 1.7-21.4%) and substantially below the thresholds that clinically mandate tissue biopsy.

The following justification has been added to the Statistical Analysis section of the revised Methods (Track Changes, lines 121-126, Revised Manuscript with Track Changes):

*"The primary analysis classified Categories IV (Suspicious for Malignancy) and V (Malignant) as test-positive, and Categories II (Benign) and III (Atypical) as test-negative, consistent with the IAC Yokohama System framework, in which Category III carries a low risk of malignancy (ROM = 6.7%; 95% CI: 0.2-31.9%) and is recommended for follow-up or repeat FNA rather than immediate surgical intervention. Two pre-specified sensitivity analyses were performed to assess the robustness of this classification scheme (S4 Table)."*

***Part 2 - Sensitivity analyses***

Two pre-specified analyses were performed on the identical dataset (n = 104; from Table 2 data):

- Sensitivity Analysis 1 (SA1, n = 89): The 15 Category III cases were excluded, retaining only cases with definitive cytological classification.
- Sensitivity Analysis 2 (SA2, n = 104): Category III was reclassified as test-positive (low-threshold scenario).

Results are summarised in the table below and presented in full in S4 Table:

| **Diagnostic Metric** | **Main Analysis (n=104) Cat IV+V = Pos** | **SA1 (n=89) Cat III excluded** | **SA2 (n=104) Cat III = Pos** |
| --- | --- | --- | --- |
| Sensitivity | 92.5% (82.1-97.9) | 94.2% (84.4-98.0) | 94.3% (84.6-98.1) |
| Specificity | 96.1% (86.5-99.5) | 94.6% (82.3-98.5) | **68.6%** (55.0-79.7)^*^ |
| PPV | 96.1% (86.5-99.5) | 96.1% (86.8-98.9) | **75.8%** (64.2-84.5)^*^ |
| NPV | 92.5% (82.1-97.9) | 92.1% (79.2-97.3) | 92.1% (79.2-97.3) |
| Accuracy | 94.2% (88.1-97.8) | 94.4% (87.5-97.6) | **81.7%** (73.2-88.0)^*^ |

** Marked reduction in SA2 reflects the clinical cost of treating atypical cytology as an actionable positive result.*

Excluding Category III cases (SA1) yields virtually identical performance metrics (sensitivity 94.2%, specificity 94.6%), confirming that the 15 atypical cases did not disproportionately inflate the primary estimates. In contrast, SA2 demonstrates that reclassifying Category III as test-positive substantially reduces specificity (96.1% → 68.6%) and PPV (96.1% → 75.8%) without a meaningful gain in sensitivity (92.5% → 94.3%), further supporting the validity of the primary classification

***Changes in the revised manuscript:***

(1) Methods - Statistical Analysis: two sentences added to justify the primary classification and pre-specify SA1/SA2, with reference to S4 Table (Track Changes, lines 121-126, Revised Manuscript with Track Changes).
(2) Results - one sentence added after Table 3 (Track Changes, line 172): "Results were consistent across two pre-specified sensitivity analyses; full details are provided in S4 Table."
(3) Supporting Information: S4 Table added. No other data, statistics, or conclusions have been modified.

**Comment 4:** *The multivariate model includes age, tumor size, and BI-RADS classification, but the BI-RADS 5 subgroup is very small: only 11 cases, including 10 malignant and 1 benign lesion. Please pay attention. At minimum, the paper should state that the nonsignificant BI-RADS 5 result should not be interpreted as evidence that BI-RADS 5 lacks clinical value.*

**Response:** We agree that the non-significant result for BI-RADS 5 in the multivariate model must not be interpreted as evidence against its clinical utility. While this caveat was present in the Discussion section of the original manuscript (lines 230-231), it was not sufficiently prominent in the Abstract or Conclusion - the sections most frequently read in isolation. Explicit cautionary statements have been added at both locations.

***Changes in the revised manuscript:***

(1) Abstract - the following sentence has been added immediately after reporting the BI-RADS 5 p-value (Track Changes):

*"However, given the small BI-RADS 5 subgroup (n = 11), this non-significant result should not be interpreted as evidence of its lack of clinical utility, as the analysis was likely underpowered for this specific variable."*

(2) Conclusion - the following sentence has been inserted before the final sentence of the Conclusion (Track Changes):

*"Of note, the non-significant result for BI-RADS 5 in the multivariate model (p = 0.540; aOR 2.10, 95% CI: 0.20-22.73) should not be interpreted as evidence that BI-RADS 5 classification lacks clinical value; rather, it reflects the limited statistical power of the small BI-RADS 5 subgroup (n = 11), which rendered this estimate susceptible to Type II error."*

The Discussion (lines 230-231), which already contained this caveat, has been retained unchanged. No data, statistics, or other conclusions have been modified.

We hope that the revised manuscript and the responses provided above are satisfactory. We remain available should the Editor or Reviewers require any further clarification.

Sincerely,

**Assoc. Prof. Dang Thanh Chung, MD, PhD**

On behalf of the co-authors

Vice Head, Department of Pathology and Forensic Medicine, Military Hospital 103

Vietnam Military Medical University, Hanoi, Vietnam

*dangthanhchung@vmmu.edu.vn*
