# Supplementary material for: Age and tumor size as independent predictors of malignancy in BI-RADS 4 and 5 breast lesions: A cross-sectional study in Vietnam
Source: PLoS One. 2026 Jul 6;21(7):e0352690. doi: 10.1371/journal.pone.0352690 (PMC13336213; doi:10.1371/journal.pone.0352690)
Supplement: Peer review history

# Author comment: Author response to Decision Letter 0

**DOI:** 10.1371/journal.pone.0352690.r001

**Transfer Alert**

This paper was transferred from another journal. As a result, its full editorial history (including decision letters, peer reviews and author responses) may not be present.

16 Sep 2025

# Peer review: Decision Letter 0

**DOI:** 10.1371/journal.pone.0352690.r002

**Authors:** Dongling Wu

19 Jan 2026

PONE-D-25-49862

Age and Tumor Size as Independent Predictors of Malignancy in BI-RADS 4 and 5 Breast Lesions: A Crosssectional Study in Vietnam

PLOS One

Dear Dr. Dang,

Thank you for submitting your manuscript to PLOS ONE. After careful consideration, we feel that it has merit but does not fully meet PLOS ONE’s publication criteria as it currently stands. Therefore, we invite you to submit a revised version of the manuscript that addresses the points raised during the review process.

- **In general, this is a very well written manuscript, however, the sample size of the study is small, making the results questionable. In this revision, I highly recommend you to include more years of data and cases to make the study reliable.**

-->Please submit your revised manuscript by Mar 05 2026 11:59PM. If you will need more time than this to complete your revisions, please reply to this message or contact the journal office at plosone@plos.org. When you're ready to submit your revision, log on to https://www.editorialmanager.com/pone/ and select the 'Submissions Needing Revision' folder to locate your manuscript file.

Please include the following items when submitting your revised manuscript:

- A letter that responds to each point raised by the academic editor and reviewer(s). You should upload this letter as a separate file labeled 'Response to Reviewers'.
- A marked-up copy of your manuscript that highlights changes made to the original version. You should upload this as a separate file labeled 'Revised Manuscript with Track Changes'.
- An unmarked version of your revised paper without tracked changes. You should upload this as a separate file labeled 'Manuscript'.

If you would like to make changes to your financial disclosure, please include your updated statement in your cover letter. Guidelines for resubmitting your figure files are available below the reviewer comments at the end of this letter.

If applicable, we recommend that you deposit your laboratory protocols in protocols.io to enhance the reproducibility of your results. Protocols.io assigns your protocol its own identifier (DOI) so that it can be cited independently in the future. For instructions see: https://journals.plos.org/plosone/s/submission-guidelines#loc-laboratory-protocols. Additionally, PLOS ONE offers an option for publishing peer-reviewed Lab Protocol articles, which describe protocols hosted on protocols.io. Read more information on sharing protocols at https://plos.org/protocols?utm_medium=editorial-email&utm_source=authorletters&utm_campaign=protocols.

We look forward to receiving your revised manuscript.

Kind regards,

Dongling Wu

Academic Editor

PLOS One

**Journal Requirements:**

1. When submitting your revision, we need you to address these additional requirements.

Please ensure that your manuscript meets PLOS ONE's style requirements, including those for file naming. The PLOS ONE style templates can be found at

https://journals.plos.org/plosone/s/file?id=wjVg/PLOSOne_formatting_sample_main_body.pdf and

https://journals.plos.org/plosone/s/file?id=ba62/PLOSOne_formatting_sample_title_authors_affiliations.pdf

2. Please include a copy of S1 Checklist. STROBE Checklist which you refer to in your text on pages 242.

3. If the reviewer comments include a recommendation to cite specific previously published works, please review and evaluate these publications to determine whether they are relevant and should be cited. There is no requirement to cite these works unless the editor has indicated otherwise.

**Additional Editor Comments:**

This is a well-written manuscript. However, one of the major drawbacks of this study is the limited sample size especially the malignancy group making the results questionable. I highly recommend the author group, in this revision, to include more cases (looking back more years' data) for a more reliable result.

[Note: HTML markup is below. Please do not edit.]

Reviewers' comments:

Reviewer's Responses to Questions

**Comments to the Author**

1. Is the manuscript technically sound, and do the data support the conclusions?

The manuscript must describe a technically sound piece of scientific research with data that supports the conclusions. Experiments must have been conducted rigorously, with appropriate controls, replication, and sample sizes. The conclusions must be drawn appropriately based on the data presented.

Reviewer #1: Partly

Reviewer #2: Yes

**********

2. Has the statistical analysis been performed appropriately and rigorously?

Reviewer #1: Yes

Reviewer #2: Yes

**********

3. Have the authors made all data underlying the findings in their manuscript fully available?

The PLOS Data policy requires authors to make all data underlying the findings described in their manuscript fully available without restriction, with rare exception (please refer to the Data Availability Statement in the manuscript PDF file). The data should be provided as part of the manuscript or its supporting information, or deposited to a public repository. For example, in addition to summary statistics, the data points behind means, medians and variance measures should be available. If there are restrictions on publicly sharing data—e.g. participant privacy or use of data from a third party—those must be specified.

Reviewer #1: No

Reviewer #2: Yes

**********

4. Is the manuscript presented in an intelligible fashion and written in standard English?

PLOS ONE does not copyedit accepted manuscripts, so the language in submitted articles must be clear, correct, and unambiguous. Any typographical or grammatical errors should be corrected at revision, so please note any specific errors here.

Reviewer #1: Yes

Reviewer #2: Yes

**********

5. Review Comments to the Author

Please use the space provided to explain your answers to the questions above. You may also include additional comments for the author, including concerns about dual publication, research ethics, or publication ethics. (Please upload your review as an attachment if it exceeds 20,000 characters)

**Reviewer #1:**  The sample size is small (104 lesions) and key subgroups particularly BI-RADS 5 lesions are underpowered as reflected by extremely wide confidence intervals in the multivariate analysis. No sample size justification or power calculation is provided, raising concerns about Type II errors and the reliability of reported associations.

**Reviewer #2:**  The manuscript is very well written, the methodology is well conducted, and the work is robust. I offer a few recommendations. Some paragraphs (especially in the Discussion) are long and could be made slightly more objective. The manuscript alternates between shorter sentences and some very long ones; a few of these could be broken up to improve readability.

I also suggest reinforcing clarity in the tables. In Table 1, the positioning of the p-values could be reorganized to make the layout visually cleaner. In Table 2, it may be useful to include the exact ROM values with their confidence intervals, should the authors wish to strengthen the presentation.

Although the limitations are already well described, the authors could consider adding: the potential bias associated with conducting the study in tertiary centers, and the possibility of interobserver variability in cytology interpretation (even though consensus among pathologists was used).

Finally, consider adding a concluding paragraph that more explicitly reinforces the clinical applicability of the findings.

**********

6. PLOS authors have the option to publish the peer review history of their article (what does this mean?). If published, this will include your full peer review and any attached files.

If you choose “no”, your identity will remain anonymous but your review may still be made public.

**Do you want your identity to be public for this peer review?**  For information about this choice, including consent withdrawal, please see our Privacy Policy.

Reviewer #1: No

Reviewer #2: No

**********

[NOTE: If reviewer comments were submitted as an attachment file, they will be attached to this email and accessible via the submission site. Please log into your account, locate the manuscript record, and check for the action link "View Attachments". If this link does not appear, there are no attachment files.]

To ensure your figures meet our technical requirements, please review our figure guidelines: https://journals.plos.org/plosone/s/figures

You may also use PLOS’s free figure tool, NAAS, to help you prepare publication quality figures: https://journals.plos.org/plosone/s/figures#loc-tools-for-figure-preparation.

NAAS will assess whether your figures meet our technical requirements by comparing each figure against our figure specifications.

# Author comment: Author response to Decision Letter 1

**DOI:** 10.1371/journal.pone.0352690.r003

29 Jan 2026

January 26, 2026

Ref: PONE-D-25-49862 Title: Age and tumor size as independent predictors of malignancy in BI-RADS 4 and 5 breast lesions: A cross-sectional study in Vietnam

Dear Dr. Dongling Wu and Reviewers,

We would like to express our sincere gratitude for the time and effort you have dedicated to reviewing our manuscript. We appreciate the insightful comments and constructive suggestions, which have helped us significantly improve the quality and rigor of our study.

We have carefully reviewed all points raised. In this revised version, we have addressed the concerns regarding sample size by providing a post-hoc power analysis and clarifying our strict inclusion criteria. We have also complied with PLOS ONE’s data policy by uploading a minimal underlying dataset (S2 Dataset).

Below is our point-by-point response to the specific comments.

RESPONSE TO JOURNAL REQUIREMENTS

1. PLOS ONE Style Requirements

Comment: Please ensure that your manuscript meets PLOS ONE's style requirements, including those for file naming.

Response: We have reviewed the PLOS ONE style templates and formatted our manuscript accordingly. Specifically, we have adjusted the headings (Level 1 in 18pt bold), figure citations, and reference style to meet the journal's guidelines.

2. STROBE Checklist

Comment: Please include a copy of S1 Checklist. STROBE Checklist which you refer to in your text.

Response: We have completed the STROBE Checklist with page numbers indicating where each item is addressed in the revised manuscript. This file has been uploaded as S1 Checklist.

3. Data Availability

Comment: “Have the authors made all data underlying the findings in their manuscript fully available?” is not sufficient.

Response: We apologize for the initial restriction. We have now prepared a de-identified minimal dataset containing the values behind the means, standard deviations, and other measures reported. This dataset complies with ethical standards for patient anonymity and has been uploaded as a Supporting Information file labeled ‘S2 Dataset’. The Data Availability Statement within the manuscript has been updated accordingly.

RESPONSE TO REVIEWER #1

Comment 1: Sample size and Statistical power

Reviewer #1: The sample size is small (104 lesions) and key subgroups particularly BI-RADS 5 lesions are underpowered as reflected by extremely wide confidence intervals in the multivariate analysis. No sample size justification or power calculation is provided, raising concerns about Type II errors and the reliability of reported associations.

Response: We thank the reviewer for this critical assessment. We agree that the sample size, particularly for the BI-RADS 5 subgroup (n=11), is a limitation. Regarding the suggestion to include more years of data, we evaluated this possibility but found it unfeasible due to our strict inclusion criteria, which required matched high-quality ultrasound-guided FNA and definitive histopathology (biopsy/surgery) for every patient to ensure diagnostic accuracy. Extending the timeframe retrospectively would have compromised data completeness and quality due to administrative limitations on older physical records.

However, to address the concern regarding Type II errors and reliability, we have performed a post-hoc power analysis based on the observed effect sizes in our study (added to Methods, Page 6-7, Lines: 113-116)

- For tumor size: The observed effect size was medium-to-large (≈ 0.64). Our analysis indicates the study achieved a statistical power of 89.5% (α=0.05) to detect this primary predictor. This suggests the finding regarding tumor size is robust.

- For patient age: The observed effect size was moderate (≈ 0.48), yielding a power of 67.2%. While slightly below the conventional 80%, the significant association (p = 0.003) suggests a detectable signal in our population.

We have also revised the Discussion section (Pages 14, Lines 219-228) to explicitly acknowledge that the BI-RADS 5 subgroup is underpowered, leading to wide confidence intervals, and that findings regarding this specific subgroup should be interpreted as preliminary.

Comment 2: Data Availability

Reviewer #1: Have the authors made all data underlying the findings in their manuscript fully available? No.

Response: As mentioned in the response to the Journal Requirements, we have now uploaded the fully anonymized minimal dataset as S2 Dataset.

RESPONSE TO REVIEWER #2

Comment 1: Methodology and Writing

Reviewer #2: The manuscript is very well written, the methodology is well conducted, and the work is robust. I offer a few recommendations.

Response: We are grateful for the reviewer’s positive evaluation of our work and the helpful recommendations to improve the manuscript’s readability.

Comment 2: Paragraph Length and Readability

Reviewer #2: Some paragraphs (especially in the Discussion) are long and could be made slightly more objective. The manuscript alternates between shorter sentences and some very long ones; a few of these could be broken up to improve readability.

Response: We have reviewed the Discussion section and broken down long paragraphs and complex sentences to improve flow and clarity, as suggested. (See changes on Pages 12, 13, 14).

Comment 3: Table Clarity

Reviewer #2: In Table 1, the positioning of the p-values could be reorganized to make the layout visually cleaner. In Table 2, it may be useful to include the exact ROM values with their confidence intervals, should the authors wish to strengthen the presentation.

Response:

- Table 1: We have reorganized the layout of Table 1. P-values are now presented in a dedicated column on the right side to improve readability and visual alignmen (Pages 8 - 9).

- Table 2: We have calculated the 95% Confidence Intervals (CI) for the Risk of Malignancy (ROM) using the Wilson score interval method (appropriate for small sample sizes). These values have been added to the last column of Table 2 (Page 9).

Comment 4: Limitations (Tertiary Centers & Interobserver Variability)

Reviewer #2: Although the limitations are already well described, the authors could consider adding: the potential bias associated with conducting the study in tertiary centers, and the possibility of interobserver variability in cytology interpretation (even though consensus among pathologists was used).

Response: We agree these are important points.

- We have reinforced the statement regarding the tertiary center bias (Page 14, Lines 218-219).

- We have added a sentence to the Limitations section acknowledging the potential for interobserver variability in cytology interpretation, despite our consensus method (Page 14, Lines 228-230).

Comment 5: Clinical Applicability

Reviewer #2: Finally, consider adding a concluding paragraph that more explicitly reinforces the clinical applicability of the findings.

Response: We have revised the Conclusion to explicitly state the clinical implication: in resource-limited settings like Vietnam, integrating Age and Tumor Size into the risk assessment of BI-RADS 4/5 lesions can help clinicians prioritize patients for urgent intervention. (Page 15, Lines 247-251).

Sincerely,

Assoc. Prof. Dang Thanh Chung, MD, PhD

On behalf of the co-authors

Vice Head, Department of Pathology and Forensic Medicine Military Hospital 103,

Vietnam Military Medical University Hanoi, Vietnam

dangthanhchung@vmmu.edu.vn

Attachment Submitted filename: Response to Reviewers.doc

# Peer review: Decision Letter 1

**DOI:** 10.1371/journal.pone.0352690.r004

**Authors:** Dongling Wu

22 May 2026

PONE-D-25-49862R1

Age and tumor size as independent predictors of malignancy in BI-RADS 4 and 5 breast lesions: A cross-sectional study in Vietnam

PLOS One

Dear Dr.  Dang,

Thank you for submitting your manuscript to PLOS ONE and thank you for your patience. After careful consideration, we feel that it has merit but does not fully meet PLOS ONE’s publication criteria as it currently stands. Therefore, we invite you to submit a revised version of the manuscript that addresses the points raised during the review process.

Please submit your revised manuscript by Jul 06 2026 11:59PM. If you will need more time than this to complete your revisions, please reply to this message or contact the journal office at plosone@plos.org. When you're ready to submit your revision, log on to https://www.editorialmanager.com/pone/ and select the 'Submissions Needing Revision' folder to locate your manuscript file.

Please include the following items when submitting your revised manuscript:

- A letter that responds to each point raised by the academic editor and reviewer(s). You should upload this letter as a separate file labeled 'Response to Reviewers'.
- A marked-up copy of your manuscript that highlights changes made to the original version. You should upload this as a separate file labeled 'Revised Manuscript with Track Changes'.
- An unmarked version of your revised paper without tracked changes. You should upload this as a separate file labeled 'Manuscript'.

If you would like to make changes to your financial disclosure, please include your updated statement in your cover letter. Guidelines for resubmitting your figure files are available below the reviewer comments at the end of this letter.

If applicable, we recommend that you deposit your laboratory protocols in protocols.io to enhance the reproducibility of your results. Protocols.io assigns your protocol its own identifier (DOI) so that it can be cited independently in the future. For instructions see: https://journals.plos.org/plosone/s/submission-guidelines#loc-laboratory-protocols. Additionally, PLOS ONE offers an option for publishing peer-reviewed Lab Protocol articles, which describe protocols hosted on protocols.io. Read more information on sharing protocols at https://plos.org/protocols?utm_medium=editorial-email&utm_source=authorletters&utm_campaign=protocols.

As the corresponding author, your ORCID iD is verified in the submission system and will appear in the published article. PLOS supports the use of ORCID, and we encourage all coauthors to register for an ORCID iD and use it as well. Please encourage your coauthors to verify their ORCID iD within the submission system before final acceptance, as unverified ORCID iDs will not appear in the published article. *Only*  the individual author can complete the verification step; PLOS staff *cannot*  verify ORCID iDs on behalf of authors.

We look forward to receiving your revised manuscript.

Kind regards,

Dongling Wu

Academic Editor

PLOS One

Journal Requirements:

If the reviewer comments include a recommendation to cite specific previously published works, please review and evaluate these publications to determine whether they are relevant and should be cited. There is no requirement to cite these works unless the editor has indicated otherwise.

Please review your reference list to ensure that it is complete and correct. If you have cited papers that have been retracted, please include the rationale for doing so in the manuscript text, or remove these references and replace them with relevant current references. Any changes to the reference list should be mentioned in the rebuttal letter that accompanies your revised manuscript. If you need to cite a retracted article, indicate the article’s retracted status in the References list and also include a citation and full reference for the retraction notice.

Reviewers' comments:

Reviewer's Responses to Questions

**Comments to the Author**

1. If the authors have adequately addressed your comments raised in a previous round of review and you feel that this manuscript is now acceptable for publication, you may indicate that here to bypass the “Comments to the Author” section, enter your conflict of interest statement in the “Confidential to Editor” section, and submit your "Accept" recommendation.

Reviewer #2: All comments have been addressed

Reviewer #3: (No Response)

**********

2. Is the manuscript technically sound, and do the data support the conclusions?

The manuscript must describe a technically sound piece of scientific research with data that supports the conclusions. Experiments must have been conducted rigorously, with appropriate controls, replication, and sample sizes. The conclusions must be drawn appropriately based on the data presented.

Reviewer #2: Yes

Reviewer #3: Yes

**********

3. Has the statistical analysis been performed appropriately and rigorously?

Reviewer #2: Yes

Reviewer #3: Yes

**********

4. Have the authors made all data underlying the findings in their manuscript fully available?

The PLOS Data policy requires authors to make all data underlying the findings described in their manuscript fully available without restriction, with rare exception (please refer to the Data Availability Statement in the manuscript PDF file). The data should be provided as part of the manuscript or its supporting information, or deposited to a public repository. For example, in addition to summary statistics, the data points behind means, medians and variance measures should be available. If there are restrictions on publicly sharing data—e.g. participant privacy or use of data from a third party—those must be specified.

Reviewer #2: Yes

Reviewer #3: Yes

**********

5. Is the manuscript presented in an intelligible fashion and written in standard English?

PLOS ONE does not copyedit accepted manuscripts, so the language in submitted articles must be clear, correct, and unambiguous. Any typographical or grammatical errors should be corrected at revision, so please note any specific errors here.

Reviewer #2: (No Response)

Reviewer #3: Yes

**********

6. Review Comments to the Author

Please use the space provided to explain your answers to the questions above. You may also include additional comments for the author, including concerns about dual publication, research ethics, or publication ethics. (Please upload your review as an attachment if it exceeds 20,000 characters)

Reviewer #2: The authors addressed all comments satisfactorily and improved the manuscript, particularly regarding the clarity of the writing and the transparency of the presented data.

Reviewer #3: The revised manuscript is clinically relevant and generally well organized. The authors have strengthened the revision by adding confidence intervals, post hoc power information, limitations related to the small BI-RADS 5 subgroup, and clearer clinical applicability.

Several points:

1. The manuscript would be stronger if the authors provided a table summarizing the histopathologic diagnoses, including benign entities, DCIS, invasive carcinoma of no special type, special histologic types if present, and whether malignant cases were invasive or in situ.

2. Similarly, if available, BI-RADS 4 should be subdivided into 4A, 4B, and 4C. Without this, the BI-RADS 4 group is heterogeneous, and the clinical meaning of comparing BI-RADS 4 versus BI-RADS 5 is limited.

3. The authors classified Categories IV and V as positive, and Categories II and III as negative for diagnostic performance calculations. This approach may underestimate the clinical uncertainty associated with “atypical” cytology. The authors should justify this decision and ideally provide sensitivity analyses: one excluding Category III cases and another treating Category III as a test-positive or indeterminate result. This would make the reported sensitivity, specificity, PPV, NPV, and accuracy more transparent.

4. The multivariate model includes age, tumor size, and BI-RADS classification, but the BI-RADS 5 subgroup is very small: only 11 cases, including 10 malignant and 1 benign lesion. Please pay attention. At minimum, the paper should state that the nonsignificant BI-RADS 5 result should not be interpreted as evidence that BI-RADS 5 lacks clinical value.

**********

7. PLOS authors have the option to publish the peer review history of their article (what does this mean?). If published, this will include your full peer review and any attached files.

If you choose “no”, your identity will remain anonymous but your review may still be made public.

**Do you want your identity to be public for this peer review?**  For information about this choice, including consent withdrawal, please see our Privacy Policy.

Reviewer #2: No

Reviewer #3: No

**********

[NOTE: If reviewer comments were submitted as an attachment file, they will be attached to this email and accessible via the submission site. Please log into your account, locate the manuscript record, and check for the action link "View Attachments". If this link does not appear, there are no attachment files.]

To ensure your figures meet our technical requirements, please review our figure guidelines: https://journals.plos.org/plosone/s/figures

You may also use PLOS’s free figure tool, NAAS, to help you prepare publication quality figures: https://journals.plos.org/plosone/s/figures#loc-tools-for-figure-preparation.

NAAS will assess whether your figures meet our technical requirements by comparing each figure against our figure specifications.

# Author comment: Author response to Decision Letter 2

**DOI:** 10.1371/journal.pone.0352690.r005

27 May 2026

May 27, 2026

Ref: PONE-D-25-49862R1

Title: Age and tumor size as independent predictors of malignancy in BI-RADS 4 and 5 breast lesions: A cross-sectional study in Vietnam

Journal: PLOS ONE

Dear Dr. Dongling Wu and Members of the Editorial Board,

We thank the Academic Editor and Reviewers for their careful evaluation and constructive comments, which have substantially strengthened the revised manuscript. We have addressed all four comments raised by Reviewer 3. A point-by-point response is provided below. All revisions are visible in the Revised Manuscript with Track Changes.

RESPONSE TO REVIEWER #2

Comment: The authors addressed all comments satisfactorily and improved the manuscript, particularly regarding the clarity of the writing and the transparency of the presented data.

Response: We thank Reviewer 2 for this positive assessment.

RESPONSE TO REVIEWER #3

We thank Reviewer 3 for the thorough and methodologically rigorous comments. Responses to each point are provided below, with corresponding changes indicated by line numbers in the Revised Manuscript with Track Changes.

Comment 1: The manuscript would be stronger if the authors provided a table summarizing the histopathologic diagnoses, including benign entities, DCIS, invasive carcinoma of no special type, special histologic types if present, and whether malignant cases were invasive or in situ.

Response: We have prepared Supplementary Table S3 (S3 Table), which provides a complete breakdown of all histopathological diagnoses for the 104 lesions, classified per the WHO Classification of Breast Tumours, 4th Edition (2019). Specifically, S3 Table:

1. Lists all benign entities individually (fibroadenoma, fibrocystic change, intraductal papilloma, mastitis/inflammatory lesion, phyllodes tumour, sclerosing adenosis, and epithelial hyperplasia, usual type), per WHO 2019 nomenclature.

2. Clearly distinguishes non-invasive from invasive malignancies: ductal carcinoma in situ (DCIS), NOS (n = 1) is listed separately under “B1. Non-invasive.”

3. Lists all special histological subtypes of invasive carcinoma: invasive carcinoma of no special type (NST; n = 47, including one case with a solid growth pattern), tubular carcinoma (n = 4), and mucinous carcinoma (n = 1), consistent with WHO 2019 nomenclature.

Changes in the revised manuscript:

(1) The following sentence has been added to the Results section, end of the 'Patient and lesion characteristics' paragraph:

"The specific histopathological diagnoses for all lesions, including individual benign entities and a distinction between in situ and invasive malignancies, are provided in S3 Table."

(2) S3 Table has been added to the Supporting Information list at the end of the manuscript. No existing text, data, or tables (Tables 1-4) have been modified.

Comment 2: Similarly, if available, BI-RADS 4 should be subdivided into 4A, 4B, and 4C. Without this, the BI-RADS 4 group is heterogeneous, and the clinical meaning of comparing BI-RADS 4 versus BI-RADS 5 is limited.

Response: The suggestion is fully justified: the ACR BI-RADS Atlas (5th ed., 2013) assigns meaningfully different risks of malignancy to each subcategory (4A: ≥2% to <10%; 4B: ≥10% to ≤50%; 4C: >50% to <95%). However, BI-RADS 4 subcategory data (4A/4B/4C) were not available in the source medical records. The study incorporated data from two military tertiary hospitals in Vietnam collected between January 2021 and September 2023, a period during which structured subcategory reporting was not consistently documented in institutional radiology reports. The binary BI-RADS 4 vs. 5 classification therefore reflects the data as recorded in the original clinical records.

We fully acknowledge that the undifferentiated BI-RADS 4 group introduces heterogeneity into the analysis and limits the clinical interpretability of the BI-RADS 4 vs. 5 comparison. This has been explicitly stated as an additional limitation in the revised Discussion section.

Changes in the revised manuscript:

The following paragraph has been added as the fourth item in the Limitations section of the Discussion (Track Changes):

"Fourth, subcategory data for BI-RADS 4 lesions (4A, 4B, and 4C) were unavailable, as structured subcategory reporting was not consistently documented in the institutional radiology records during the retrospective data collection period (2021-2023). This limits the granularity of the BI-RADS analysis, as the three BI-RADS 4 subcategories carry substantially different risks of malignancy (4A: ≥2% to <10%; 4B: ≥10% to ≤50%; 4C: >50% to <95%). Future prospective studies should incorporate BI-RADS 4 subcategory classification to enable a more refined analysis of imaging-based malignancy predictors within this heterogeneous group."

No data, statistics, or existing conclusions have been modified.

Comment 3: The authors classified Categories IV and V as positive, and Categories II and III as negative for diagnostic performance calculations. This approach may underestimate the clinical uncertainty associated with “atypical” cytology. The authors should justify this decision and ideally provide sensitivity analyses: one excluding Category III cases and another treating Category III as a test-positive or indeterminate result. This would make the reported sensitivity, specificity, PPV, NPV, and accuracy more transparent.

Response: We agree with this assessment and have responded with: (1) a formal justification of the primary classification scheme, incorporated into the Methods section; and (2) two pre-specified sensitivity analyses, presented in S4 Table.

Part 1 - Justification of the primary classification (Cat IV+V = Positive)

The decision to classify Categories IV and V as test-positive and Categories II and III as test-negative was made a priori, following the IAC Yokohama System framework (Field AS et al. Acta Cytol. 2019;63:257-273), in which Category III (Atypical) carries a low risk of malignancy and is recommended for repeat FNA or close follow-up rather than immediate surgical intervention. In our cohort, Category III yielded a ROM of 6.7% (95% CI: 0.2-31.9%), which is statistically comparable to Category II (ROM 7.9%; 95% CI: 1.7-21.4%) and substantially below the thresholds that clinically mandate tissue biopsy.

The following justification has been added to the Statistical Analysis section of the revised Methods (Track Changes, lines 121-126, Revised Manuscript with Track Changes):

"The primary analysis classified Categories IV (Suspicious for Malignancy) and V (Malignant) as test-positive, and Categories II (Benign) and III (Atypical) as test-negative, consistent with the IAC Yokohama System framework, in which Category III carries a low risk of malignancy (ROM = 6.7%; 95% CI: 0.2-31.9%) and is recommended for follow-up or repeat FNA rather than immediate surgical intervention. Two pre-specified sensitivity analyses were performed to assess the robustness of this classification scheme (S4 Table)."

Part 2 - Sensitivity analyses

Two pre-specified analyses were performed on the identical dataset (n = 104; from Table 2 data):

- Sensitivity Analysis 1 (SA1, n = 89): The 15 Category III cases were excluded, retaining only cases with definitive cytological classification.

- Sensitivity Analysis 2 (SA2, n = 104): Category III was reclassified as test-positive (low-threshold scenario).

Results are summarised in the table below and presented in full in S4 Table:

Diagnostic Metric Main Analysis (n=104) Cat IV+V = Pos SA1 (n=89) Cat III excluded SA2 (n=104) Cat III = Pos

Sensitivity 92.5% (82.1-97.9) 94.2% (84.4-98.0) 94.3% (84.6-98.1)

Specificity 96.1% (86.5-99.5) 94.6% (82.3-98.5) 68.6% (55.0-79.7)*

PPV 96.1% (86.5-99.5) 96.1% (86.8-98.9) 75.8% (64.2-84.5)*

NPV 92.5% (82.1-97.9) 92.1% (79.2-97.3) 92.1% (79.2-97.3)

Accuracy 94.2% (88.1-97.8) 94.4% (87.5-97.6) 81.7% (73.2-88.0)*

* Marked reduction in SA2 reflects the clinical cost of treating atypical cytology as an actionable positive result.

Excluding Category III cases (SA1) yields virtually identical performance metrics (sensitivity 94.2%, specificity 94.6%), confirming that the 15 atypical cases did not disproportionately inflate the primary estimates. In contrast, SA2 demonstrates that reclassifying Category III as test-positive substantially reduces specificity (96.1% → 68.6%) and PPV (96.1% → 75.8%) without a meaningful gain in sensitivity (92.5% → 94.3%), further supporting the validity of the primary classification

Changes in the revised manuscript:

(1) Methods - Statistical Analysis: two sentences added to justify the primary classification and pre-specify SA1/SA2, with reference to S4 Table (Track Changes, lines 121-126, Revised Manuscript with Track Changes).

(2) Results - one sentence added after Table 3 (Track Changes, line 172): "Results were consistent across two pre-specified sensitivity analyses; full details are provided in S4 Table."

(3) Supporting Information: S4 Table added. No other data, statistics, or conclusions have been modified.

Comment 4: The multivariate model includes age, tumor size, and BI-RADS classification, but the BI-RADS 5 subgroup is very small: only 11 cases, including 10 malignant and 1 benign lesion. Please pay attention. At minimum, the paper should state that the nonsignificant BI-RADS 5 result should not be interpreted as evidence that BI-RADS 5 lacks clinical value.

Response: We agree that the non-significant result for BI-RADS 5 in the multivariate model must not be interpreted as evidence against its clinical utility. While this caveat was present in the Discussion section of the original manuscript (lines 230-231), it was not sufficiently prominent in the Abstract or Conclusion - the sections most frequently read in isolation. Explicit cautionary statements have been added at both locations.

Changes in the revised manuscript:

(1) Abstract - the following sentence has been added immediately after reporting the BI-RADS 5 p-value (Track Changes):

"However, given the small BI-RADS 5 subgroup (n = 11), this non-significant result should not be interpreted as evidence of its lack of clinical utility, as the analysis was likely underpowered for this specific variable."

(2) Conclusion - the following sentence has been inserted before the final sentence of the Conclusion (Track Changes):

"Of note, the non-significant result for BI-RADS 5 in the multivariate model (p = 0.540; aOR 2.10, 95% CI: 0.20-22.73) should not be interpreted as evidence that BI-RADS 5 classification lacks clinical value; rather, it reflects the limited statistical power of the small BI-RADS 5 subgroup (n = 11), which rendered this estimate susceptible to Type II error."

The Discussion (lines 230-231), which already contained this caveat, has been retained unchanged. No data, statistics, or other conclusions have been modified.

We hope that the revised manuscript and the responses provided above are satisfactory. We remain available should the Editor or Reviewers require any further clarification.

Sincerely,

Assoc. Prof. Dang Thanh Chung, MD, PhD

On behalf of the co-authors

Vice Head, Department of Pathology and Forensic Medicine, Military Hospital 103

Vietnam Military Medical University, Hanoi, Vietnam

dangthanhchung@vmmu.edu.vn

Attachment Submitted filename: Response_to_Reviewers_auresp_2.doc

# Editor report: Decision Letter 2

**DOI:** 10.1371/journal.pone.0352690.r006

**Authors:** Dongling Wu

15 Jun 2026

Age and tumor size as independent predictors of malignancy in BI-RADS 4 and 5 breast lesions: A cross-sectional study in Vietnam

PONE-D-25-49862R2

Dear Dr. Dang,

We’re pleased to inform you that your manuscript has been judged scientifically suitable for publication and will be formally accepted for publication once it meets all outstanding technical requirements.

Within one week, you’ll receive an e-mail detailing the required amendments. When these have been addressed, you’ll receive a formal acceptance letter and your manuscript will be scheduled for publication.

An invoice will be generated when your article is formally accepted. Please note, if your institution has a publishing partnership with PLOS and your article meets the relevant criteria, all or part of your publication costs will be covered. Please make sure your user information is up-to-date by logging into Editorial Manager at Editorial Manager® and clicking the ‘Update My Information' link at the top of the page. For questions related to billing, please contact billing support.

If your institution or institutions have a press office, please notify them about your upcoming paper to help maximize its impact. If they’ll be preparing press materials, please inform our press team as soon as possible -- no later than 48 hours after receiving the formal acceptance. Your manuscript will remain under strict press embargo until 2 pm Eastern Time on the date of publication. For more information, please contact onepress@plos.org.

Kind regards,

Dongling Wu

Academic Editor

PLOS One

Additional Editor Comments (optional):

The author has fully addressed the reviewer's comments, and the study reaches publication level.

Reviewers' comments:

# Editor report: Acceptance letter

**DOI:** 10.1371/journal.pone.0352690.r007

**Authors:** Dongling Wu

PONE-D-25-49862R2

PLOS One

Dear Dr. Dang,

I'm pleased to inform you that your manuscript has been deemed suitable for publication in PLOS One. Congratulations! Your manuscript is now being handed over to our production team.

At this stage, our production department will prepare your paper for publication. This includes ensuring the following:

* All references, tables, and figures are properly cited

* All relevant supporting information is included in the manuscript submission,

* There are no issues that prevent the paper from being properly typeset

You will receive further instructions from the production team, including instructions on how to review your proof when it is ready. Please keep in mind that we are working through a large volume of accepted articles, so please give us a few days to review your paper and let you know the next and final steps.

Lastly, if your institution or institutions have a press office, please let them know about your upcoming paper now to help maximize its impact. If they'll be preparing press materials, please inform our press team within the next 48 hours. Your manuscript will remain under strict press embargo until 2 pm Eastern Time on the date of publication. For more information, please contact onepress@plos.org.

You will receive an invoice from PLOS for your publication fee after your manuscript has reached the completed accept phase. If you receive an email requesting payment before acceptance or for any other service, this may be a phishing scheme. Learn how to identify phishing emails and protect your accounts at https://explore.plos.org/phishing.

If we can help with anything else, please email us at customercare@plos.org.

Thank you for submitting your work to PLOS ONE and supporting open access.

Kind regards,

PLOS ONE Editorial Office Staff

on behalf of

Dr. Dongling Wu

Academic Editor

PLOS One
